# Emergency Medicine Residency Applicant Characteristics Associated with Measured Adverse Outcomes During Residency

**DOI:** 10.5811/westjem.2017.11.35007

**Published:** 2017-12-21

**Authors:** Jesse Bohrer-Clancy, Leslie Lukowski, Lisa Turner, Ilene Staff, Shawn London

**Affiliations:** * University of Connecticut, Department of Emergency Medicine, Farmington, Connecticut; † Hartford Hospital, Department of Emergency Medicine, Hartford, Connecticut; ‡ Hartford Hospital, Proposal Design and Statistical Analysis, Hartford, Connecticut

## Abstract

**Introduction:**

Negative outcomes in emergency medicine (EM) programs use a disproportionate amount of educational resources to the detriment of other residents. We sought to determine if any applicant characteristics identifiable during the selection process are associated with negative outcomes during residency.

**Methods:**

Primary analysis consisted of looking at the association of each of the descriptors including resident characteristics and events during residency with a composite measure of negative outcomes. Components of the negative outcome composite were any formal remediation, failure to complete residency, or extension of residency.

**Results:**

From a dataset of 260 residents who completed their residency over a 19-year period, 26 (10%) were osteopaths and 33 (13%) were international medical school graduates A leave of absence during medical school (p <.001), failure to send a thank-you note (p=.008), a failing score on United States Medical Licensing Examination Step I (p=.002), and a prior career in health (p=.034) were factors associated with greater likelihood of a negative outcome. All four residents with a “red flag” during their medicine clerkships experienced a negative outcome (p <.001).

**Conclusion:**

“Red flags” during EM clerkships, a leave of absence during medical school for any reason and failure to send post-interview thank-you notes may be associated with negative outcomes during an EM residency.

## INTRODUCTION

The application process for emergency medicine (EM) residencies is designed to not only allow the applicant to evaluate different programs, but to also allow residency programs to determine which residents will be a good fit for their program. Residencies examine various applicant characteristics and try to assess not only which applicants will fit in but which will also hopefully thrive in their program. This screening process is also a key part of attempting to predict which applicants may experience difficulty during training, whether it is due to inadequate medical knowledge, poor patient care or issues with professionalism. If these applicants make it through the application process and matriculate, they can cause a disproportionate drain on the residency’s teaching and leadership resources, create interpersonal difficulties among the residents or create service hardships through lost resident work effort relating to a leave of absence or dismissal from the program.

Attempts at predicting success in an EM program through the analysis of applicant characteristics has been done in a number of previous studies^1^–^6^ as well as in obstetrics/gynecology^7^ and orthopedic surgical residencies,^8^ among others. Surgery programs have found a weak correlation between USMLE scores and certain tests of gross manual dexterity.^9^

However, the reverse question has not been as well studied and we were unable to identify studies that specifically target applicant characteristics related to poor performance in EM residency. Corrective action during a residency, such as formal letters of deficiency (LoD) for performance or professionalism and letters of reprimand (LoR) for issues dealing with professionalism, are considered negative outcomes since they typically precede an extension, and residents subject to discipline may be at risk of dismissal, leaving a residency with few options to replace the lost individual.

We initiated a retrospective analysis of applicant and resident data in the past 19 years of records currently held by the University of Connecticut (UConn) EM residency program to determine if there are characteristics in the residency application that are associated with negative outcomes during residency.

## METHODS

We analyzed the dataset to see if there was an association between a variety of different applicant characteristics and any measured negative outcome, including LoRs, LoDs, extension of residency (EXT) and failure to finish residency in our program (DNF). This study received a waiver from the

UConn Institutional Review Board as a quality improvement study. This was a purely investigational study designed to elicit details about the evaluation of future residency applicants through the retrospective analysis of existing data from previous years of archived data on residents who matched at the U Conn EM residency.

All data were manually collected by the program coordinators (L.L. & L.T.) from the Electronic Residency Application Service (ERAS) applications in the matriculated resident personnel files held by UConn’s EM residency. The coordinators already had access to the data used in this study and assigned each application a unique random identifier to de-identify residents for the dataset. Applicant details such as gender, medical school attended and year of graduation from medical school or residency were removed, and the data was anonymized using two different randomization schemes known only to the coordinators to prevent possible identification of residents from the research database. We input the applicant data into a Microsoft Excel spreadsheet (Redmond, WA). Negative outcomes, if present, were input from resident files and are listed in Table 1.

We used IBM SPSSv 21 (Armonk, NY) for analysis of the different variables. The primary analysis consisted of looking at the association of each of the descriptors with the composite measure of negative outcome using chi-square tests of proportion or Fisher’s exact test when cell frequencies were low (Appendix A). The list of the descriptors used were as follows: those with a Doctor of Medicine (M.D.) degree vs. those with a Doctor of Osteopathic Medicine (D.O.) degree; the presence of a prior career; the presence of prior healthcare experience; U.S. vs. international medical school graduate (IMG); whether a leave of absence was taken during medical school; failure to transmit medical transcripts to ERAS; whether a post-interview thank-you note was sent; and the presence of “red flags” during EM clerkship (defined as marked deficiencies in letters of recommendation from the clerkship director or written comments from attending or resident physicians from UConn medical school clerkship rotations).

**Population Health Research Capsule**

What do we already know about this issue?

Prior research relied on subjective endpoints of resident outcomes but did not identify features of emergency medicine applicants associated with objective negative outcomes in residency.

What was the research question?

We sought to determine if any EM applicant characteristics are associated with negative outcomes during residency.

What was the major finding of the study?

Leave of absence and the lack of a thank-you note sent to the program were found to be independent predictors of negative outcomes.

How does this improve population health?

These findings may help residencies identify which EM applicants are at risk of compromising residency resources devoted to patient care and negatively impacting population health.

United States Medical Licensing Examination (USMLE) Step 1 and Step 2 scores, average interview score, and the resident’s position on the final rank list are continuous variables and were evaluated by Wilcoxon ranked-sum test, comparing the subgroups defined by the composite measure. As these factors are likely to be interrelated, we used a multivariate approach to determine which factors independently were related to a negative outcome. A logistic regression model was created using those factors that showed a significant result with the outcome variable (Appendix B). (Because information on “red flags” was available for only a small subgroup, we eliminated them from the multivariate analysis.)

## RESULTS

The population of the dataset was 260 residents, 26 of whom were D.O.s (10%) and 33 (13%) IMGs. (All of the IMGs were allopaths but for the purposes of reporting will be listed as an IMG). There were 49 residents with one or more of the negative outcomes, representing 18.8% of the total 260 residents over the past 19 years. There were 19 LoRs, 23 LoDs for any reason, 13 residents had to extend their residency, and eight did not finish the program.

Among the 23 LoDs, 16 (10 M.D., four IMG, two D.O.) did not have a specification listed; three (two M.D., one IMG) were for patient care; three (two M.D., one IMG) were for medical knowledge; and eight (six M.D., two IMG) were for professionalism. Some letters were combined so that one letter may have contained two elements; e.g., a single letter noting deficiencies in both patient care and medical knowledge were counted as separate in this study.

Of the 13 residents who had to extend their residency training, nine were M.D.s, three IMGs and one was a D.O. Two extensions were due to problems with patient care (one M.D., one IMG); two for medical knowledge (one M.D., one IMG) and six were due to lack of professionalism (four M.D., two IMG). Of the eight residents who did not finish the program, all were allopaths; two of the eight were IMG, but this was not statistically significant. Interestingly, of the eight residents who did not finish three had prior healthcare experience, although this was not statistically significant.

The single factor most associated with a negative outcome was a prior leave of absence in medical school for any reason. The data kept by the program did not specify the reason for the leave of absence, just that a leave had occurred. Residents with a leave of absence in medical school for any reason had an increased likelihood of a negative outcome in 94.1%% vs. 5.9% for the residency in general, p<0.001.

Thank-you notes appeared to have an inverse correlation with negative outcomes. The residency recorded whether or not a thank-you note had been sent after the applicant was interviewed. Residents who did not send a thank-you note after their interviews had an increased likelihood of any negative outcome (25.5% vs 12.4%, p=0.008).

Residents who received a failing score on the USMLE Step I exam during medical school were significantly more likely to have had a negative outcome during training (46.2% vs. 17.5%; p= 0.020). “Red flags” during the applicant’s EM clerkship had a very strong correlation with the negative outcomes we tracked in this study. A specific notation was made in the file of an applicant if a resident or attending working with the applicant at one of the UConn clinical sites or a letter of recommendation from another program raised grave concerns about the student’s performance in the emergency department or professionalism. Residents with a “red flag” in their application had a 100% vs. 6% (p=<0.001) chance of a negative outcome during residency compared to residents who had no “red flags.”

A logistic regression (Appendix C) predicting the composite of any negative outcome was run with dichotomous predictors for whether a thank-you letter was sent, a leave of absence was taken during medical school, the applicant had a prior career in healthcare, and a failing score on the USMLE Step 1 test entered simultaneously. Leave of absence (p.<.001) and the lack of a thank-you note (p =.004) were found to be independent predictors of negative outcome (Table 2).

## DISCUSSION

This study found that many of the discriminators that are part of the ERAS residency application did not have an association with the negative outcomes in our dataset. Our analysis revealed that failure to transmit transcripts during the ERAS process and prior non-healthcare experience had no bearing on negative outcomes in residency. While we expected there to be an association between negative outcomes in residency and “red flags” during the EM clerkship, the negative associations related to prior heathcare experience was an unexpected finding, as our program looks upon prior experience as a positive applicant attribute. The negative association with a failure to send a thank-you note after the interview was also an unexpected finding.

While a failing grade on the USMLE Step I exam was associated with negative outcomes during training this does not seem to be an unexpected finding, as individuals who had medical knowledge deficits in medical school would intuitively seem to be more likely to require a formal remediation plan (termed a LoD at UConn) during residency, similar to the findings of Wagner et al.^10^ We found that D.O.s had a decreased chance of having negative outcomes in our residency, but this finding may be due to selection bias given the relatively small number of D.O.s in our program. Similarly, while IMG status did not confer a statistically significant chance of negative outcomes during residency, a relatively small number of IMGs were present in our dataset. Areas for further study include more detailed analysis of clerkship grades, medical school class rank and the rank of the applicant’s medical school. Other studies have found that IMGs have a lower rate of residency completion.^11^

## LIMITATIONS

This study was a retrospective analysis of the applicant data of matriculated residents at a single EM residency program and may not be generalizable to other EM residencies or other specialties. The dataset was also limited in certain respects due to some USMLE Step scores only being recorded as pass or fail. This pass/fail scoring prevented us from analyzing a delta between each of the tests to determine whether improvement or worsening of board scores between the steps was significant. Analysis of USMLE scores was also limited by the fact that while most residents had these scores recorded, some osteopathic residents had only sat for the Comprehensive Osteopathic Medical Licensing Examination.

Furthermore, the lack of standardization of medical school grades and reporting of medical school class rank was also problematic. The lack of a universal presence of Alpha Omega Alpha (AOA) chapters at allopathic medical schools or Sigma Sigma Phi at osteopathic medical schools precluded analysis of nomination as an attribute. The lack of an objective measure of ranking medical schools themselves did not give us an objective measure by which to assess a correlation between medical school reputation and resident outcomes.

## CONCLUSION

Our analysis revealed that “red flags” during emergency medicine clerkships, a leave of absence during medical school for any reason and failure to send post-interview thank-you notes were all associated with negative outcomes during a three-year emergency medicine residency.

## Supplementary Information







## Figures and Tables

**Table 1:** Applicant characteristics entered into research database.

Descriptor
USMLE Step 1
USMLE Step 2CK
USMLE Step 2CS
USMLE Step 3
COMLEX
Leave during medical school
“Red flags” during EM clerkship
Failure to transmit medical school transcripts to ERAS
Surgical clerkship grade
Pediatric clerkship grade
OB/GYN clerkship grade
Psychiatry clerkship grade
Overall GPA
Class rank
Medical school rank
Undergraduate major
MD vs DO
IMG (yes/no)
Prior career (yes/no)
Prior healthcare experience (yes/no)
Average interview score
Program director score
Post interview thank you note sent (yes/no)
Number of final rank list

*USMLE*, United States Medical Licensing Examination; *CK*, clinical knowledge; *CS*, clinical skills; *COMLEX*, Comprehensive Osteopathic Medical Licensing Examination; *EM,* emergency medicine; *ERAS,* Electronic Residency Application Service; *OB/GYN,* obstetrics and gynecology; *GPA*, grade point average; *MD*, doctor of medicine; *DO*, Doctor of Osteopathic Medicine; *IMG*, international medical graduate.

**Table 2:** Resident characteristics and factors’ effect on composite measure of negative outcomes.

Factor (measurement)	No negative outcomes (N=211)	One or more negative outcomes (N=49)	P value
Degree (N, %)			
Yes	188 (80.3)	46 (19.7)	.432
No	23 (88.5)	3 (11.5)	
Prior health experience			
Yes	62 (80.5)	15 (19.5)	.798
No	149 (81.9)	33 (18.1)	
Prior career in health			
Yes	48 (72.7)	18 (27.3)	.034
No	163 (84.5)	30 (15.5)	
Foreign medical school			
Yes	24 (72.7)	9 (27.3)	.185
No	187 (82.4)	40 (17.6)	
Transcript			
Yes	5 (62.5)	3 (37.5)	.162
No	206 (82.4)	44 (17.6)	
Thank you sent			
Yes	127 (87.6)	18 (12.1)	.008
No	79 (74.5)	27 (25.5)	
Red Flags (N = 71)			
Yes	0 (0)	4 (100)	<.001
No	63 (94.0)	4 (6.0)	
Leave of absence			
Yes	1 (5.9)	16 (94.1)	<.001
No	210 (86.4)	33 (13.6)	
USMLE Step 1 failing score			
Yes	7 (53.8)	6 (46.2)	.020
No	203 (82.5)	43 (17.5)	
USMLE Step 2 failing score			
Yes	37 (74.0)	13 (26.0)	.130
No	174 (83.3)	35 (16.7)	
Filler rank (median, IQR)	54 (26,74)	67 (49,81)	.064
Interview score (median, IQR)	3.5 (3,4)	3.5 (3,4)	.189

*MD*, doctor of medicine; *DO*, Doctor of Osteopathic Medicine; *USMLE*, United States Medical Licensing Examination; *IQR*, interquartile range.

**Table 3:** Predicting composite measure of negative outcomes: simultaneous logistic regression.

Predictor	Odds ratio (OR)	(95% CI for OR)	P value
Sending thank you note	3.16	1.44 – 6.91	0.004
Leave of absence	98.05	11.62 – 827.71	< 0.001
USMLW Step I failing score	1.44	0.28 – 7.46	.662
Prior career in health	2.064	0.91 – 4.678	.082
