# Supplementary material for: Emergency Medicine Residency Applicant Characteristics Associated with Measured Adverse Outcomes During Residency
Source: West J Emerg Med. 2017 Dec 21;19(1):106–11. doi: 10.5811/westjem.2017.11.35007 (PMC5785175; doi:10.5811/westjem.2017.11.35007)
Supplement: Supplementary file 1 [file wjem-19-106-s001.pdf]

```
DATASET ACTIVATE DataSet2.
* redoing all just on composite.
get file ='Q:\Outcomes Projects\Emergency Dept\Bohrer resident survey\Residen
tSituation analysis 10-23-17.sav'.
* SEEING RELATIONSHIPS BETWEEN POSSIBLE FACTORS AND OUTCOMES.

* prior to acceptance.
* degree prior career and prior health experience.
crosstabs degree  priorExperHealth PriorCareer FMG by composite4Neg
     /cells =count row
     /statistics = chisq.
```

Crosstabs

Q:\Outcomes Projects\Emergency Dept\Bohrer resident survey\ResidentSituation analysis 10-23-17.sav

Case Processing Summary

	Cases					
	Valid		Missing		Total	
	N	Percent	N	Percent	N	Percent
degree * composite4Neg DNF, extension, LOD or LOR probation during residency	**260**	**100.0%**	**0**	**.0%**	**260**	**100.0%**
priorExperHealth * composite4Neg DNF, extension, LOD or LOR probation during residency	**259**	**99.6%**	**1**	**.4%**	**260**	**100.0%**
PriorCareer * composite4Neg DNF, extension, LOD or LOR probation during residency	**259**	**99.6%**	**1**	**.4%**	**260**	**100.0%**
FMG foreign medical school graduate * composite4Neg DNF, extension, LOD or LOR probation during residency	**260**	**100.0%**	**0**	**.0%**	**260**	**100.0%**

degree * composite4Neg DNF, extension, LOD or LOR probation during residency

Crosstab

			composite4Neg DNF, extension, LOD or LOR probation during residency		
			0 no	1 yes	Total
degree	1 MD	Count	**188**	**46**	**234**
		% within degree	**80.3%**	**19.7%**	**100.0%**
	2 DO	Count	**23**	**3**	**26**
		% within degree	**88.5%**	**11.5%**	**100.0%**
Total		Count	**211**	**49**	**260**
		% within degree	**81.2%**	**18.8%**	**100.0%**

Chi-Square Tests

	Value	df	Asymp. Sig. (2-sided)	Exact Sig. (2-sided)	Exact Sig. (1-sided)
Pearson Chi-Square	**1.009**[a]	**1**	**.315**		
Continuity Correction [b]	**.548**	**1**	**.459**		
Likelihood Ratio	**1.122**	**1**	**.289**		
Fisher's Exact Test				**.432**	**.237**
Linear-by-Linear Association	**1.005**	**1**	**.316**		
N of Valid Cases	**260**				

a. 1 cells (25.0%) have expected count less than 5. The minimum expected count is 4.90.
b. Computed only for a 2x2 table

priorExperHealth * composite4Neg DNF, extension, LOD or LOR probation during residency

Crosstab

			composite4Neg DNF, extension, LOD or LOR probation during residency		
			0 no	1 yes	Total
priorExperHealth	0 no	Count	**149**	**33**	**182**
		% within priorExperHealth	**81.9%**	**18.1%**	**100.0%**
	1 yes	Count	**62**	**15**	**77**
		% within priorExperHealth	**80.5%**	**19.5%**	**100.0%**
Total		Count	**211**	**48**	**259**
		% within priorExperHealth	**81.5%**	**18.5%**	**100.0%**

Chi-Square Tests

	Value	df	Asymp. Sig. (2-sided)	Exact Sig. (2-sided)	Exact Sig. (1-sided)
Pearson Chi-Square	**.065**[a]	**1**	**.798**		
Continuity Correction [b]	**.006**	**1**	**.936**		
Likelihood Ratio	**.065**	**1**	**.799**		
Fisher's Exact Test				**.861**	**.462**
Linear-by-Linear Association	**.065**	**1**	**.799**		
N of Valid Cases	**259**				

a. 0 cells (.0%) have expected count less than 5. The minimum expected count is 14.27.
b. Computed only for a 2x2 table

PriorCareer * composite4Neg DNF, extension, LOD or LOR probation during residency

Crosstab

			composite4Neg DNF, extension, LOD or LOR probation during residency		
			0 no	1 yes	Total
PriorCareer	.0 no	Count	**163**	**30**	**193**
		% within PriorCareer	**84.5%**	**15.5%**	**100.0%**
	1.0 yes	Count	**48**	**18**	**66**
		% within PriorCareer	**72.7%**	**27.3%**	**100.0%**
Total		Count	**211**	**48**	**259**
		% within PriorCareer	**81.5%**	**18.5%**	**100.0%**

Chi-Square Tests

	Value	df	Asymp. Sig. (2-sided)	Exact Sig. (2-sided)	Exact Sig. (1-sided)
Pearson Chi-Square	**4.481**[a]	**1**	**.034**		
Continuity Correction [b]	**3.738**	**1**	**.053**		
Likelihood Ratio	**4.208**	**1**	**.040**		
Fisher's Exact Test				**.043**	**.029**
Linear-by-Linear Association	**4.464**	**1**	**.035**		
N of Valid Cases	**259**				

a. 0 cells (.0%) have expected count less than 5. The minimum expected count is 12.23.
b. Computed only for a 2x2 table

FMG foreign medical school graduate * composite4Neg DNF, extension, LOD or LOR probation during residency

Crosstab

			composite4Neg DNF, extension, LOD or LOR probation during residency		
			0 no	1 yes	Total
FMG foreign medical school graduate	.0 no	Count	**187**	**40**	**227**
		% within FMG foreign medical school graduate	**82.4%**	**17.6%**	**100.0%**
	1.0 yes	Count	**24**	**9**	**33**
		% within FMG foreign medical school graduate	**72.7%**	**27.3%**	**100.0%**
Total		Count	**211**	**49**	**260**
		% within FMG foreign medical school graduate	**81.2%**	**18.8%**	**100.0%**

Chi-Square Tests

	Value	df	Asymp. Sig. (2-sided)	Exact Sig. (2-sided)	Exact Sig. (1-sided)
Pearson Chi-Square	**1.755**[a]	**1**	**.185**		
Continuity Correction [b]	**1.180**	**1**	**.277**		
Likelihood Ratio	**1.617**	**1**	**.204**		
Fisher's Exact Test				**.231**	**.139**
Linear-by-Linear Association	**1.748**	**1**	**.186**		
N of Valid Cases	**260**				

a. 0 cells (.0%) have expected count less than 5. The minimum expected count is 6.22.
b. Computed only for a 2x2 table

```
* other issues and actions.
crosstabs noTranscript thankyousent Redflags by composite4Neg
     /cells =count row
     /statistics = chisq.
```

Crosstabs

Q:\Outcomes Projects\Emergency Dept\Bohrer resident survey\ResidentSituation analysis 10-23-17.sav

Case Processing Summary

	Cases					
	Valid		Missing		Total	
	N	Percent	N	Percent	N	Percent
noTranscript transcript submission failure * composite4Neg DNF, extension, LOD or LOR probation during residency	**258**	**99.2%**	**2**	**.8%**	**260**	**100.0%**
thankyousent * composite4Neg DNF, extension, LOD or LOR probation during residency	**251**	**96.5%**	**9**	**3.5%**	**260**	**100.0%**
Redflags * composite4Neg DNF, extension, LOD or LOR probation during residency	**71**	**27.3%**	**189**	**72.7%**	**260**	**100.0%**

noTranscript transcript submission failure * composite4Neg DNF, extension, LOD or LOR probation during residency

Crosstab

			composite4Neg DNF, extension, LOD or LOR probation during residency		
			0 no	1 yes	Total
noTranscript transcript submission failure	0 no, no transcript issue	Count	**206**	**44**	**250**
		% within noTranscript transcript submission failure	**82.4%**	**17.6%**	**100.0%**
	1 yes, transcript not sent	Count	**5**	**3**	**8**
		% within noTranscript transcript submission failure	**62.5%**	**37.5%**	**100.0%**
Total		Count	**211**	**47**	**258**
		% within noTranscript transcript submission failure	**81.8%**	**18.2%**	**100.0%**

Chi-Square Tests

	Value	df	Asymp. Sig. (2-sided)	Exact Sig. (2-sided)	Exact Sig. (1-sided)
Pearson Chi-Square	**2.061**[a]	**1**	**.151**		
Continuity Correction[b]	**.941**	**1**	**.332**		
Likelihood Ratio	**1.707**	**1**	**.191**		
Fisher's Exact Test				**.162**	**.162**
Linear-by-Linear Association	**2.053**	**1**	**.152**		
N of Valid Cases	**258**				

a. 1 cells (25.0%) have expected count less than 5. The minimum expected count is 1.46.
b. Computed only for a 2x2 table

thankyousent * composite4Neg DNF, extension, LOD or LOR probation during residency

Crosstab

			composite4Neg DNF, extension, LOD or LOR probation during residency		
			0 no	1 yes	Total
thankyousent	.0 no	Count	**79**	**27**	**106**
		% within thankyousent	**74.5%**	**25.5%**	**100.0%**
	1.0 yes	Count	**127**	**18**	**145**
		% within thankyousent	**87.6%**	**12.4%**	**100.0%**
Total		Count	**206**	**45**	**251**
		% within thankyousent	**82.1%**	**17.9%**	**100.0%**

Chi-Square Tests

	Value	df	Asymp. Sig. (2-sided)	Exact Sig. (2-sided)	Exact Sig. (1-sided)
Pearson Chi-Square	**7.096**[a]	**1**	**.008**		
Continuity Correction [b]	**6.236**	**1**	**.013**		
Likelihood Ratio	**7.016**	**1**	**.008**		
Fisher's Exact Test				**.012**	**.006**
Linear-by-Linear Association	**7.068**	**1**	**.008**		
N of Valid Cases	**251**				

a. 0 cells (.0%) have expected count less than 5. The minimum expected count is 19.00.
b. Computed only for a 2x2 table

Redflags * composite4Neg DNF, extension, LOD or LOR probation during residency

Crosstab

			composite4Neg DNF, extension, LOD or LOR probation during residency		
			0 no	1 yes	Total
Redflags	.0 no	Count	**63**	**4**	**67**
		% within Redflags	**94.0%**	**6.0%**	**100.0%**
	1.0 yes	Count	**0**	**4**	**4**
		% within Redflags	**.0%**	**100.0%**	**100.0%**
Total		Count	**63**	**8**	**71**
		% within Redflags	**88.7%**	**11.3%**	**100.0%**

Chi-Square Tests

	Value	df	Asymp. Sig. (2-sided)	Exact Sig. (2-sided)	Exact Sig. (1-sided)
Pearson Chi-Square	**33.381**[a]	**1**	**.000**		
Continuity Correction [b]	**24.638**	**1**	**.000**		
Likelihood Ratio	**19.691**	**1**	**.000**		
Fisher's Exact Test				**.000**	**.000**
Linear-by-Linear Association	**32.910**	**1**	**.000**		
N of Valid Cases	**71**				

a. 2 cells (50.0%) have expected count less than 5. The minimum expected count is .45.
b. Computed only for a 2x2 table

```
* tests.
crosstabs USME1PF USME2PF by composite4Neg
     /cells =count row
```

```
/statistics = chisq.
```

Crosstabs

Q:\Outcomes Projects\Emergency Dept\Bohrer resident survey\ResidentSituation analysis 10-23-17.sav

Case Processing Summary

	Cases					
	Valid		Missing		Total	
	N	Percent	N	Percent	N	Percent
USME1PF * composite4Neg DNF, extension, LOD or LOR probation during residency	**259**	**99.6%**	**1**	**.4%**	**260**	**100.0%**
USME2PF * composite4Neg DNF, extension, LOD or LOR probation during residency	**259**	**99.6%**	**1**	**.4%**	**260**	**100.0%**

USME1PF * composite4Neg DNF, extension, LOD or LOR probation during residency

Crosstab

			composite4Neg DNF, extension, LOD or LOR probation during residency		
			0 no	1 yes	Total
USME1PF	0	Count	**7**	**6**	**13**
		% within USME1PF	**53.8%**	**46.2%**	**100.0%**
	1	Count	**203**	**43**	**246**
		% within USME1PF	**82.5%**	**17.5%**	**100.0%**
Total		Count	**210**	**49**	**259**
		% within USME1PF	**81.1%**	**18.9%**	**100.0%**

Chi-Square Tests

	Value	df	Asymp. Sig. (2-sided)	Exact Sig. (2-sided)	Exact Sig. (1-sided)
Pearson Chi-Square	**6.618**[a]	**1**	**.010**		
Continuity Correction[b]	**4.881**	**1**	**.027**		
Likelihood Ratio	**5.310**	**1**	**.021**		
Fisher's Exact Test				**.020**	**.020**
Linear-by-Linear Association	**6.593**	**1**	**.010**		
N of Valid Cases	**259**				

a. 1 cells (25.0%) have expected count less than 5. The minimum expected count is 2.46.
b. Computed only for a 2x2 table

USME2PF * composite4Neg DNF, extension, LOD or LOR probation during residency

Crosstab

			composite4Neg DNF, extension, LOD or LOR probation during residency		
			0 no	1 yes	Total
USME2PF	0	Count	**37**	**13**	**50**
		% within USME2PF	**74.0%**	**26.0%**	**100.0%**
	1	Count	**174**	**35**	**209**
		% within USME2PF	**83.3%**	**16.7%**	**100.0%**
Total		Count	**211**	**48**	**259**
		% within USME2PF	**81.5%**	**18.5%**	**100.0%**

Chi-Square Tests

	Value	df	Asymp. Sig. (2-sided)	Exact Sig. (2-sided)	Exact Sig. (1-sided)
Pearson Chi-Square	**2.288**[a]	**1**	**.130**		
Continuity Correction[b]	**1.716**	**1**	**.190**		
Likelihood Ratio	**2.142**	**1**	**.143**		
Fisher's Exact Test				**.156**	**.098**
Linear-by-Linear Association	**2.279**	**1**	**.131**		
N of Valid Cases	**259**				

a. 0 cells (.0%) have expected count less than 5. The minimum expected count is 9.27.
b. Computed only for a 2x2 table

```
* finished, no extension no letters either kind.
sort cases by composite4Neg.
split file by composite4Neg.
frequencies variables = fillerrank AvgInterviewScore
     /format = notable
      /statistics = mean stdev median minimum maximum
      /percentiles 25 75.
```

Frequencies

Q:\Outcomes Projects\Emergency Dept\Bohrer resident survey\ResidentSituation analysis 10-23-17.sav

Statistics

composite4Neg DNF, extension, LO...			FillerRank	AvgInterviewScore
0 no	N	Valid	**180**	**206**
		Missing	**31**	**5**
	Mean		**52.822**	**3.5864**
	Median		**54.000**	**3.5000**
	Std. Deviation		**31.4284**	**.66368**
	Minimum		**1.0**	**.10**
	Maximum		**133.0**	**5.00**
	Percentiles	25	**26.000**	**3.0000**
		75	**74.000**	**4.0000**
1 yes	N	Valid	**35**	**43**
		Missing	**14**	**6**
	Mean		**63.200**	**3.4477**
	Median		**67.000**	**3.5000**
	Std. Deviation		**27.6882**	**.71871**
	Minimum		**3.0**	**2.00**
	Maximum		**111.0**	**5.00**
	Percentiles	25	**49.000**	**3.0000**
		75	**81.000**	**4.0000**

```
split file off.
npar tests m-w fillerrank AvgInterviewScore by composite4Neg (0,1).
```

NPar Tests

Q:\Outcomes Projects\Emergency Dept\Bohrer resident survey\ResidentSituation analysis 10-23-17.sav

Mann-Whitney Test

Ranks

	composite4Neg DNF...	N	Mean Rank	Sum of Ranks
FillerRank	0 no	**180**	**104.53**	**18,816.00**
	1 yes	**35**	**125.83**	**4,404.00**
	Total	**215**		
AvgInterviewScore	0 no	**206**	**127.68**	**26,301.50**
	1 yes	**43**	**112.17**	**4,823.50**
	Total	**249**		

Test Statistics[a]

	FillerRank	AvgInterviewScore
Mann-Whitney U	**2,526.000**	**3,877.500**
Wilcoxon W	**18,816.000**	**4,823.500**
Z	**-1.853**	**-1.313**
Asymp. Sig. (2-tailed)	**.064**	**.189**

a. Grouping Variable: composite4Neg DNF, extension, LOD or LOR probation during residency

```
* once in residency.
```

```
crosstabs leaveYN USME3PF by composite4Neg
     /cells =count row
     /statistics = chisq.
```

Crosstabs

Q:\Outcomes Projects\Emergency Dept\Bohrer resident survey\ResidentSituation analysis 10-23-17.sav

Case Processing Summary

	Cases					
	Valid		Missing		Total	
	N	Percent	N	Percent	N	Percent
leaveYN medical leave taken * composite4Neg DNF, extension, LOD or LOR probation during residency	**260**	**100.0%**	**0**	**.0%**	**260**	**100.0%**
USME3PF * composite4Neg DNF, extension, LOD or LOR probation during residency	**240**	**92.3%**	**20**	**7.7%**	**260**	**100.0%**

leaveYN medical leave taken * composite4Neg DNF, extension, LOD or LOR probation during residency

Crosstab

			composite4Neg DNF, extension, LOD or LOR probation during residency		Total
			0 no	1 yes	
leaveYN medical leave taken	0 no	Count	**210**	**33**	**243**
		% within leaveYN medical leave taken	**86.4%**	**13.6%**	**100.0%**
	1 yes	Count	**1**	**16**	**17**
		% within leaveYN medical leave taken	**5.9%**	**94.1%**	**100.0%**
Total		Count	**211**	**49**	**260**
		% within leaveYN medical leave taken	**81.2%**	**18.8%**	**100.0%**

Chi-Square Tests

	Value	df	Asymp. Sig. (2-sided)	Exact Sig. (2-sided)	Exact Sig. (1-sided)
Pearson Chi-Square	**67.382**[a]	**1**	**.000**		
Continuity Correction [b]	**62.219**	**1**	**.000**		
Likelihood Ratio	**50.992**	**1**	**.000**		
Fisher's Exact Test				**.000**	**.000**
Linear-by-Linear Association	**67.123**	**1**	**.000**		
N of Valid Cases	**260**				

a. 1 cells (25.0%) have expected count less than 5. The minimum expected count is 3.20.
b. Computed only for a 2x2 table

USME3PF * composite4Neg DNF, extension, LOD or LOR probation during residency

Crosstab

			composite4Neg DNF, extension, LOD or LOR probation during residency		
			0 no	1 yes	Total
USME3PF	0	Count	**4**	**0**	**4**
		% within USME3PF	**100.0%**	**.0%**	**100.0%**
	1	Count	**190**	**46**	**236**
		% within USME3PF	**80.5%**	**19.5%**	**100.0%**
Total		Count	**194**	**46**	**240**
		% within USME3PF	**80.8%**	**19.2%**	**100.0%**

Chi-Square Tests

	Value	df	Asymp. Sig. (2-sided)	Exact Sig. (2-sided)	Exact Sig. (1-sided)
Pearson Chi-Square	**.965**[a]	**1**	**.326**		
Continuity Correction [b]	**.117**	**1**	**.733**		
Likelihood Ratio	**1.718**	**1**	**.190**		
Fisher's Exact Test				**1.000**	**.424**
Linear-by-Linear Association	**.961**	**1**	**.327**		
N of Valid Cases	**240**				

a. 2 cells (50.0%) have expected count less than 5. The minimum expected count is .77.
b. Computed only for a 2x2 table

```
split file by composite4Neg.
frequencies variables = ExamPGY1 ExamPGY2 ExamPGY3
     /format = notable
      /statistics = mean stdev median minimum maximum
      /percentiles 25 75.
```

Frequencies

Q:\Outcomes Projects\Emergency Dept\Bohrer resident survey\ResidentSituation analysis 10-23-17.sav

Statistics

composite4Neg DNF, extension, LO...			ExamPGY1	ExamPGY2	ExamPGY3
0 no	N	Valid	**183**	**165**	**160**
		Missing	**28**	**46**	**51**
	Mean		**.6972**	**.7583**	**.8024**
	Median		**.6900**	**.7600**	**.8000**
	Std. Deviation		**.06772**	**.05419**	**.04727**
	Minimum		**.50**	**.58**	**.64**
	Maximum		**.88**	**.90**	**.93**
	Percentiles	25	**.6500**	**.7250**	**.7700**
		75	**.7400**	**.7950**	**.8400**
1 yes	N	Valid	**44**	**39**	**34**
		Missing	**5**	**10**	**15**
	Mean		**.6609**	**.7244**	**.7674**
	Median		**.6500**	**.7200**	**.7600**
	Std. Deviation		**.07582**	**.06609**	**.06435**
	Minimum		**.53**	**.61**	**.61**
	Maximum		**.84**	**.89**	**.90**
	Percentiles	25	**.6100**	**.6700**	**.7175**
		75	**.7100**	**.7700**	**.8125**

```
split file off.
npar tests m-w ExamPGY1 ExamPGY2 ExamPGY3 by composite4Neg (0,1).
```

NPar Tests

Q:\Outcomes Projects\Emergency Dept\Bohrer resident survey\ResidentSituation analysis 10-23-17.sav

Mann-Whitney Test

Ranks

	composite4Neg DNF...	N	Mean Rank	Sum of Ranks
ExamPGY1	0 no	**183**	**120.67**	**22,082.50**
	1 yes	**44**	**86.26**	**3,795.50**
	Total	**227**		
ExamPGY2	0 no	**165**	**109.24**	**18,025.00**
	1 yes	**39**	**73.97**	**2,885.00**
	Total	**204**		
ExamPGY3	0 no	**160**	**103.34**	**16,535.00**
	1 yes	**34**	**70.00**	**2,380.00**
	Total	**194**		

Test Statistics[a]

	ExamPGY1	ExamPGY2	ExamPGY3
Mann-Whitney U	**2,805.500**	**2,105.000**	**1,785.000**
Wilcoxon W	**3,795.500**	**2,885.000**	**2,380.000**
Z	**-3.124**	**-3.361**	**-3.151**
Asymp. Sig. (2-tailed)	**.002**	**.001**	**.002**

a. Grouping Variable: composite4Neg DNF, extension, LOD or LOR probation during residency
