# Supplementary material for: Emergency Medicine Residency Applicant Characteristics Associated with Measured Adverse Outcomes During Residency
Source: West J Emerg Med. 2017 Dec 21;19(1):106–11. doi: 10.5811/westjem.2017.11.35007 (PMC5785175; doi:10.5811/westjem.2017.11.35007)
Supplement: Supplementary file 2 [file wjem-19-106-s002.pdf]

```
DATASET ACTIVATE DataSet1.
get file ='Q:\Outcomes Projects\Emergency Dept\Bohrer resident survey\Residen
tSituation analysis 10-23-17.sav'.

* predicting the composite measure.

LOGISTIC REGRESSION VARIABLES composite4Neg
  /METHOD=ENTER thankyousent USME1pf  leaveYN priorcareer
  /CONTRAST (thankyousent)=Indicator
 /CONTRAST (USME1pf)=Indicator (1)
 /CONTRAST (priorCareer)=Indicator (1)
 /CONTRAST (leaveYN)=Indicator (1)
  /PRINT=CI(95).
```

Logistic Regression

Q:\Outcomes Projects\Emergency Dept\Bohrer resident survey\ResidentSituation analysis 10-23-17.sav

Case Processing Summary

Unweighted Cases[a]		N	Percent
Selected Cases	Included in Analysis	250	96.2
	Missing Cases	10	3.8
	Total	260	100.0
Unselected Cases		0	.0
Total		260	100.0

a. If weight is in effect, see classification table for the total number of cases.

Dependent Variable Encoding

Original Value	Internal Value
0 no	0
1 yes	1

Categorical Variables Codings

			Parameter coding
		Frequency	(1)
PriorCareer	.0 no	185	.000
	1.0 yes	65	1.000
USME1PF	0	12	.000
	1	238	1.000
leaveYN medical leave taken	0 no	236	.000
	1 yes	14	1.000
thankyousent	.0 no	106	1.000
	1.0 yes	144	.000

Block 0: Beginning Block

Classification Table[a,b]

	Observed		Predicted: composite4Neg DNF, extension, LOD or LOR probation during residency: 0 no	1 yes	Percentage Correct
Step 0	composite4Neg DNF, extension, LOD or LOR probation during residency	0 no	**205**	**0**	**100.0**
		1 yes	**45**	**0**	**.0**
	Overall Percentage				**82.0**

a. Constant is included in the model.
b. The cut value is .500

Variables in the Equation

		B	S.E.	Wald	df	Sig.	Exp(B)
Step 0	Constant	**-1.516**	**.165**	**84.845**	**1**	**.000**	**.220**

Variables not in the Equation

			Score	df	Sig.
Step 0	Variables	thankyousent(1)	**6.960**	**1**	**.008**
		USME1PF(1)	**4.783**	**1**	**.029**
		leaveYN(1)	**56.304**	**1**	**.000**
		PriorCareer(1)	**5.590**	**1**	**.018**
	Overall Statistics		**66.121**	**4**	**.000**

Block 1: Method = Enter

Omnibus Tests of Model Coefficients

		Chi-square	df	Sig.
Step 1	Step	**53.640**	**4**	**.000**
	Block	**53.640**	**4**	**.000**
	Model	**53.640**	**4**	**.000**

Model Summary

Step	-2 Log likelihood	Cox & Snell R Square	Nagelkerke R Square
1	**182.057[a]**	**.193**	**.316**

a. Estimation terminated at iteration number 5 because parameter estimates changed by less than .001.

Classification Table[a]

	Observed		Predicted: composite4Neg DNF, extension, LOD or LOR probation during residency: 0 no	1 yes	Percentage Correct
Step 1	composite4Neg DNF, extension, LOD or LOR probation during residency	0 no	**204**	**1**	**99.5**
		1 yes	**32**	**13**	**28.9**
	Overall Percentage				**86.8**

a. The cut value is .500

Variables in the Equation

		B	S.E.	Wald	df	Sig.	Exp(B)	95% C.I.for EXP(B)	
								Lower	Upper
Step 1[a]	thankyousent(1)	**1.150**	**.399**	**8.297**	**1**	**.004**	**3.159**	**1.444**	**6.908**
	USME1PF(1)	**-.366**	**.838**	**.191**	**1**	**.662**	**.693**	**.134**	**3.583**
	leaveYN(1)	**4.586**	**1.088**	**17.751**	**1**	**.000**	**98.053**	**11.616**	**827.706**
	PriorCareer(1)	**.725**	**.417**	**3.016**	**1**	**.082**	**2.064**	**.911**	**4.677**
	Constant	**-2.326**	**.902**	**6.656**	**1**	**.010**	**.098**		

a. Variable(s) entered on step 1: thankyousent, USME1PF, leaveYN, PriorCareer.
